# Species Differences in Paraoxonase Mediated Hydrolysis of Several Organophosphorus Insecticide Metabolites

**DOI:** 10.1155/2015/470189

**Published:** 2015-02-15

**Authors:** Russell L. Carr, Mary Beth Dail, Howard W. Chambers, Janice E. Chambers

**Affiliations:** ^1^Department of Basic Sciences, Center for Environmental Health Sciences, College of Veterinary Medicine, Mississippi State University, P.O. Box 6100, Mississippi State, MS 39762-6100, USA; ^2^Department of Biochemistry, Molecular Biology, Entomology and Plant Pathology, Center for Environmental Health Sciences, Mississippi State University, Mississippi State, MS 39762, USA

## Abstract

Paraoxonase (PON1) is a calcium dependent enzyme that is capable of hydrolyzing organophosphate anticholinesterases. PON1 activity is present in most mammals and previous research established that PON1 activity differs depending on the species. These studies mainly used the organophosphate substrate paraoxon, the active metabolite of the insecticide parathion. Using serum PON1 from different mammalian species, we compared the hydrolysis of paraoxon with the hydrolysis of the active metabolites (oxons) of two additional organophosphorus insecticides, methyl parathion and chlorpyrifos. Paraoxon hydrolysis was greater than that of methyl paraoxon, but the level of activity between species displayed a similar pattern. Regardless of the species tested, the hydrolysis of chlorpyrifos-oxon was significantly greater than that of paraoxon or methyl paraoxon. These data indicate that chlorpyrifos-oxon is a better substrate for PON1 regardless of the species. The pattern of species differences in PON1 activity varied with the change in substrate to chlorpyrifos-oxon from paraoxon or methyl paraoxon. For example, the sex difference observed here and reported elsewhere in the literature for rat PON1 hydrolysis of paraoxon was not present when chlorpyrifos-oxon was the substrate.

## 1. Introduction

Organophosphorus (OP) insecticides are applied in agricultural situations for insect control and have served as the active ingredients in many household insecticides. Although their use has been restricted in many countries, they are still heavily used in many developing nations of the world [1]. In these areas, there is significant potential for the unlabelled use of agricultural grade insecticides for pest control in homes and barns with concomitant risk to companion and farm animals.

These OP insecticides exert their neurotoxic action in pests through the inhibition of acetylcholinesterase (ChE: acetylcholine hydrolase, EC 3.1.1.7), a serine esterase that degrades the widely distributed neurotransmitter acetylcholine (ACh). Significant inhibition of ChE activity can lead to the accumulation of ACh causing hyperexcitation in the central nervous system and at neuromuscular junctions. This can lead to disruption of normal physiological functioning. However, many of the OP insecticides are phosphorothionates which are not very potent inhibitors of ChE. In order to be toxic, the phosphorothionates must be converted to their active metabolites or oxons through the action of cytochrome P450. In addition to inhibiting ChE and other enzymes, the oxons are a nonphysiological substrate for paraoxonase 1 (PON1; EC 3.1.8.1), an esterase classified by its ability to hydrolyze paraoxon, the active metabolite of the insecticide parathion [2, 3].

Using diethyl p-nitrophenyl diethyl phosphate (paraoxon or E600) as a substrate, Aldridge [2, 3] first reported that distinct differences in terms of PON activity exist in the sera of various species. Others verified these species differences using paraoxon [4–7] and also diisopropyl phosphorofluoridate (DFP) [6]. Within the OP insecticide class, there are large variations in chemical structure. Previous work in rats [8, 9] and humans [10] indicates that activity differences exist for different oxons. However, it is not clear if these activity differences exist across multiple species. The present study compared the activity of PON1 from the sera of several mammalian species towards the oxons of three structurally different organophosphorus insecticides, chlorpyrifos, parathion, and methyl parathion.

## 2. Materials and Methods

### 2.1. Tissue and Biochemicals

Rabbit, pig, goat, cow, sheep, and horse sera were purchased from Sigma Chemical Company (St. Louis, MO). Three differing batches of sera were purchased with each assay conducted on a different batch of sera. Male and female rat sera were collected from an Association for Assessment and Accreditation of Laboratory Animal Care-accredited Sprague-Dawley-derived rat colony housed at the College of Veterinary Medicine, Mississippi State University. LabDiet rodent chow and tap water were freely available in this temperature-controlled environment (22 ± 2°C) with a 12 h dark-light cycle with lights on between 0700 and 1900 h. The Mississippi State University Animal Care and Use Committee approved all procedures. Analytical grade paraoxon, methyl paraoxon, and chlorpyrifos-oxon (>99%) were synthesized as previously described [11]. All other biochemicals and reagents were obtained from Sigma Chemical Company (St. Louis, MO).

### 2.2. PON1 Activities

PON1 activity was measured spectrophotometrically using our modification [9] of Furlong et al. [12–14]. The incubation mixture (1 mL) consisted of serum (5 *μ*L/mL for chlorpyrifos-oxon hydrolysis and 20 *μ*L/mL for paraoxon and methyl paraoxon), 0.05 M Tris-HCl buffer (pH 7.4), and either 1 mM CaCl_2_ (to stimulate the PON1 activity) or 1 mM EDTA (to chelate calcium, thereby preventing PON1 activity). The reaction was initiated by the addition of substrate (320 *μ*M chlorpyrifos-oxon, 5 mM paraoxon, or 5 mM methyl paraoxon in ethanol, final concentrations) and incubated for 15 min with shaking at 37°C. For chlorpyrifos-oxon hydrolysis, the reaction was terminated by the addition of 250 *μ*L of 2% sodium dodecyl sulfate and absorbance was read at 315 nm. For paraoxon and methyl paraoxon hydrolysis, the reactions were terminated by the addition of 250 *μ*L of a mixture of 2% sodium dodecyl sulfate and 2% Tris-base and absorbance was read at 400 nm. Differences between the CaCl_2_ fortified and the EDTA samples were used to correct for non-PON1 hydrolysis. For each substrate, PON1 activities were calculated as nmoles product formed min^−1 ^mg protein^−1^.

### 2.3. Statistical Analysis

The Shapiro-Wilk test was used to check the normality of the residuals and the homoscedasticity of the data. Nonnormal distribution of PON1 activity was indicated (*P* ≤ 0.05) and data were log-transformed prior to further analysis using the SAS statistical package [15]. Data were analyzed using the PROC MIXED procedure [16]. The model identified significant differences between species, OP, and species × OP interactions. Mean separation was performed by the least significant difference. The criterion for significance was set at *P* ≤ 0.05.

## 3. Results

Overall, there was a significant effect of species (*F*_7,48_ = 404.15, *P* ≤ 0.0001) and OP (*F*_2,48_ = 5245.39, *P* ≤ 0.0001). There was also a significant species × OP interaction (*F*_14,48_ = 42.44, *P* ≤ 0.0001). Thus, means were separated based on the species × OP interaction.

When paraoxon was used as the substrate, rabbit serum exhibited the highest activity of all species (Table 1). Female rat serum had significantly higher activity than male rat serum which was similar to that of goat serum. The activity in sheep serum was significantly lower than goat but significantly higher than cow, horse, and pig serum which were all statistically similar.

When chlorpyrifos-oxon was used as the substrate, rabbit serum again exhibited the highest activity of all the species (Table 1). While being significantly lower than that of rabbit, the activity in sera of female and male rats, goat, and sheep was all statistically similar. Lower activities were observed in pig, cow, and horse in decreasing order and these were statistically different from one another.

When methyl paraoxon was used as a substrate, rabbit serum also exhibited the highest activity (Table 1). Female rat serum had significantly higher activity than male rat serum which was similar to that of goat serum. The activity in goat serum was similar to that of sheep which was similar to that of horse and cow. Pig had the lowest activity towards methyl paraoxon but was statistically similar to horse and cow.

With respect to substrate affinity as measured based on activities, chlorpyrifos-oxon was a much better substrate than paraoxon and methyl paraoxon in all species (Table 1). Paraoxon was a significantly better substrate than methyl paraoxon in all species except horse, cow, and pig whose activity levels were statistically similar for paraoxon and methyl paraoxon.

## 4. Discussion

Regardless of the oxon used as substrate, rabbit serum contained higher OP hydrolysis activity. This greater ability of rabbit serum to hydrolyze OPs relative to other species was expected as it has been frequently reported in the literature [3–7]. While there is a lack of information about most species, rabbit serum PON1 has been reported to bind calcium more tightly and with more stability than human serum PON1 [17]. These two characteristics have been suggested to be partially responsible for the greater activity of rabbit serum in combination with potential quantitative (numbers of enzyme molecules per volume serum) and qualitative (catalytic turnover) differences. It has been demonstrated in humans that serum PON1 quantitative and qualitative differences are affected by single nucleotide polymorphisms in the gene's coding [12, 18] and promoter regions [19]. Gaidukov et al. [20] reported that the rabbit PON1 has a lysine (K) at amino acid position 192 which is similar in size and shape to the arginine (R) found in the human PON1 allozyme which has been [21] associated with faster hydrolysis of paraoxon. It is possible that a portion of the species differences may be due to similar variations, but many species do not possess polymorphisms.

Using paraoxon and methyl paraoxon as substrates, female rat serum possesses greater hydrolytic activity than does male rat serum. This sex difference in activity has been reported for both mice [22, 23] and rats [24, 25]. Serum PON1 is synthesized in the liver and both female mice and rats have higher levels of PON1 mRNA in their liver as compared to their male counterparts [26, 27], suggesting that the higher activity is merely a result of higher enzyme levels. In addition, the majority of serum PON1 is associated with high density lipoprotein (HDL) that contains apolipoprotein A-I (apoA-I) and the association of PON1 with apoA-I functions to stabilize enzyme activity [28]. Female rats possess higher levels of serum apoA-I and it has been proposed that this is an additional factor in the higher serum PON1 activity observed in females [24]. This sex difference in PON1 activity has been reported in a few other species including humans [29, 30] and baboons [31] and, in most cases, paraoxon was utilized as a substrate. However, when chlorpyrifos-oxon was used as the substrate, the sex difference in activity was no longer present. While this was unexpected, the absence of sex differences using chlorpyrifos-oxon as a substrate has been previously reported in mice [32].

The basis for this lack of sex differences with respect to substrate utilized is unclear. In a human study, Winnier et al. [33, 34] reported that genes other than PON1 can contribute to the sex-related variation in PON1 activity in humans. In the population studied, there were genetic sex differences located on chromosome 17 associated with sex differences in PON1 activity in the absence of any differences in the catalytic structure of PON1. It is possible that a similar situation exists in rodents, but this is unknown. Cheng and Klaassen [23] showed that androgens and male-pattern growth hormone decreased PON1 expression in male mouse livers, most probably by the activation of STAT5b which may have a response element within the mouse* PON1* promoter region. In addition, hormonal supplementation (progesterone and estradiol) of female mice for 21 days increased the levels of serum PON1 activity suggesting a role of estrogen in the higher activity of females [35]. In an* in vivo* system, addition of estradiol to a cell-based assay designed to measure cell-associated PON1 activity resulted in an increase in PON1 activity but no change in the levels of PON1 mRNA or protein, suggesting that the estrogen was enhancing the activity by stabilizing the catalytic conformation of PON1 either by inducing posttranslational modification of the protein or through inducing a factor that associates with PON1 [36]. The latter situation could explain sex differences in the activity of PON1 and partially explain the substrate-specific sex differences.

If sex differences in posttranslational modification of PON1 could slightly influence the conformation of the catalytic site, both sex and substrate differences could be present. Paraoxon and chlorpyrifos-oxon differ in structure only in their leaving group moieties with paraoxon having paranitrophenol and chlorpyrifos-oxon having trichloropyridinol. Previous cholinesterase inhibition studies with these two compounds demonstrated that the difference in the attraction of the leaving group is the basis for the different rates of binding to the enzyme [37]. A similar situation appears to exist for PON1. It has been demonstrated that slight changes in the leaving group can drastically change the *k*_cat_ (the catalytic constant for the conversion of substrate to product) for PON1 [38]. Thus, how well the leaving group is attracted to the enzyme can control its orientation into the active site and hence its catalysis. In this situation, slight differences in changes in structure in the area that attracts the leaving group can alter the rate catalysis. It may be that this difference is not detectable with very good substrates, such as chlorpyrifos-oxon whose structure has an excellent fit between enzyme and leaving group. In contrast, the differences in PON1 between sexes only become evident when a substrate has a better fit for PON1 of one sex than the other.

In conclusion, species differences exist in PON1 activity, but these differences are not merely due to greater levels of PON1. If the level of enzyme in each species was the determining factor, the pattern of activity with respect to species would be similar across substrates. However, species that differ significantly in activity with the substrates paraoxon and methyl paraoxon become similar when chlorpyrifos-oxon is used as a substrate. There are also many studies that provide solid evidence for sex-specific effects on PON1 activity in rodents [22, 24–27]. In contrast, our findings, observing that these sex-specific differences are detectable with some substrates but not detectable with others, lead us to hypothesize that the differences are possibly due to sex-specific effects on either posttranslational modification of the catalytic structure of PON1 or the induction of other factors that play roles in the activity of PON1.

## Figures and Tables

**Table 1:** Serum paraoxonase 1 specific activities (nmoles min^−1^ mg protein^−1^) from several mammalian species using different organophosphates as substrates.

	Chlorpyrifos-oxon	Paraoxon	Methyl paraoxon
Rabbit	141.479 ± 17.685^A^	54.424 ± 6.803^A^	7.530 ± 0.423^A^
Female rat	68.738 ± 5.756^B^	9.944 ± 1.020^B^	3.294 ± 0.317^B^
Goat	68.473 ± 2.497^B^	5.738 ± 0.343^C^	1.623 ± 0.139^CD^
Sheep	66.806 ± 0.451^B^	3.843 ± 0.061^D^	0.937 ± 0.080^DE^
Male rat	64.955 ± 0.944^B^	5.821 ± 0.202^C^	2.045 ± 0.183^C^
Pig	41.201 ± 2.126^C^	0.182 ± 0.033^EA∗^	0.099 ± 0.030^F∗^
Cow	30.754 ± 1.419^D^	0.770 ± 0.066^E∗^	0.321 ± 0.050^EF∗^
Horse	20.986 ± 0.299^E^	0.449 ± 0.016^E∗^	0.575 ± 0.146^EF∗^

Values presented as mean ± SEM (*n* = 3).

Values within each column (compound) not followed by the same capital letter are significantly different (*P* ≤ 0.05).

Within each row (species), values followed by an asterisk are statistically similar to one another. Absence of an asterisk indicates a significant difference (*P* ≤ 0.05) from all of the other values with that species.
